# Cost-effectiveness analysis of domiciliary topical sevoflurane for painful leg ulcers

**DOI:** 10.1371/journal.pone.0257494

**Published:** 2021-09-20

**Authors:** Carmen Selva-Sevilla, F. Dámaso Fernández-Ginés, Manuel Cortiñas-Sáenz, Manuel Gerónimo-Pardo

**Affiliations:** 1 Department of Applied Economics, Faculty of Economics, University of Castilla La Mancha, Albacete, Spain; 2 Department of Hospital Pharmacy, Torrecárdenas Hospital Complex, Almería, Spain; 3 Unit of Pain—Department of Anesthesiology, Torrecárdenas Hospital Complex, Almería, Spain; 4 Department of Anesthesiology, Complejo Hospitalario Universitario, Albacete, Spain; Universidad Nacional Autonoma de Nicaragua Leon, NICARAGUA

## Abstract

**Objectives:**

The general anesthetic sevoflurane is being repurposed as a topical analgesic for painful chronic wounds. We conducted a Bayesian cost-effectiveness analysis (CEA) comparing the addition of domiciliary topical sevoflurane to conventional analgesics (SEVOFLURANE, n = 38) versus conventional analgesics alone (CONVENTIONAL, n = 26) for the treatment of nonrevascularizable painful leg ulcers in an outpatient Pain Clinic of a Spanish tertiary hospital.

**Methods:**

We used real-world data collected from charts to conduct this CEA from a public healthcare perspective and with a one-year time horizon. Costs of analgesics, visits and admissions were considered, expressed in €2016. Analgesic effectiveness was measured with SPID (Sum of Pain Intensity Difference). A Bayesian regression model was constructed, including “treatment” and baseline characteristics for patients (“arterial hypertension”) and ulcers (“duration”, “number”, “depth”, “pain”) as covariates. The findings were summarized as a cost-effectiveness plane and a cost-effectiveness acceptability curve. One-way sensitivity analyses, a re-analysis excluding those patients who died or suffered from leg amputation, and an extreme scenario analysis were conducted to reduce uncertainty.

**Results:**

Compared to CONVENTIONAL, SEVOFLURANE was associated with a 46% reduction in costs, and the mean incremental effectiveness (28.15±3.70 effectiveness units) was favorable to SEVOFLURANE. The estimated probability for SEVOFLURANE being dominant was 99%. The regression model showed that costs were barely influenced by any covariate, whereas effectiveness was noticeably influenced by “treatment”. All sensitivity analyses showed the robustness of the model, even in the extreme scenario analysis against SEVOFLURANE.

**Conclusions:**

SEVOFLURANE was dominant over CONVENTIONAL as it was less expensive and much more effective.

## Introduction

Chronic wounds are a major health problem. In a recent systematic review of the literature, chronic wounds of mixed etiologies showed a pooled prevalence of 2.21 per 1000 population; among them, chronic leg ulcers were by far the most frequent type with an estimated prevalence of 1.51 per 1000 population [1].

The available information strongly suggests that chronic wounds represent a great economic burden [1–4]. In a recent systematic review of the literature, a global mean cost of US$ 23300 per patient and year was found (adjusted to 2015 US$), considering a health-care public payer perspective; concerning leg ulcers, and under these same assumptions, the mean cost per patient was US$ 11503 [2].

Among the symptoms of chronic wounds, pain was judged by patients as “the worst symptom and the cause of enormous suffering” [5]. Thus, the treatment of painful chronic leg ulcers should be oriented not only towards healing but also towards alleviating pain [6]. Analgesic palliative treatment becomes particularly relevant for ulcers not expected to heal, such as nonrevascularizable vascular leg ulcers [6]. Conventional analgesic drugs remain the mainstay for analgesic treatment of painful chronic wounds, mainly applying a combination of nonsteroidal anti-inflammatory drugs and adjuvants, but some refractory patients need to be treated with the specialized techniques provided by Pain Clinics. Even patients treated in these specialized settings are at risk of developing undesired effects related to analgesic drugs or complications secondary to invasive techniques [6–8].

The best way to prevent these adverse events and complications—and to reduce the associated economic burden—would be to treat painful chronic wounds with useful topical analgesics [8]. Sevoflurane is a well-known ether-derivative inhalational general anesthetic. In recent years, an emerging body of evidence has reported on the analgesic properties of sevoflurane when it is irrigated on the bed of painful leg ulcers [9–18] and wounds of several etiologies [19–21], making it an interesting new alternative for the treatment of leg ulcers.

Concerning economic evaluations in the field of chronic wounds, healing has frequently been selected as the main indicator of effectiveness, followed by the wound-free period and assessments of quality of life [22, 23], but pain has been barely considered. In the context of a randomized clinical trial, a cost-effectiveness analysis (CEA) comparing usual care for chronic leg ulcers versus a new model of community nursing care was conducted; in addition to the number of ulcers healed, the reduction in pain score was also chosen as a primary health outcome [24]. Except for this study, and as far as we know, CEAs focused on the assessment of how pain reduction could reduce the economic burden derived from painful chronic wounds are lacking [22].

As stated before, sevoflurane is showing a great clinical effectiveness when employed as a topical analgesic. However, economic evaluations of this new analgesic alternative have not been conducted so far. The main goal of this study was to conduct a CEA comparing conventional analgesics alone to treatment including domiciliary topical sevoflurane in addition to conventional analgesics for patients suffering from nonrevascularizable chronic painful leg ulcers who were referred to a Pain Clinic. This study could help healthcare decision-makers to better place sevoflurane as a topical analgesic, based on both clinical and economic criteria.

## Methods

### Background

Patients suffering from refractory pain caused by nonrevascularizable vascular chronic leg ulcers were referred to a Pain Clinic for specialized pain management. Usual analgesic treatment was mainly based on conventional systemic analgesic drugs (non-steroidal anti-inflammatory drugs, opioids, and adjuvants). In addition to conventional analgesics, patients not presenting any safety concern (mainly not having small children living in the same house, and not suffering from any mental disorders), were offered to be treated with *off-label* domiciliary topical sevoflurane following a specific protocol. Details about this protocol are provided in the S1 File.

### Study design, setting, ethical issues, and sample size

This retrospective observational cohort study was conducted in a specialized Pain Clinic of a Spanish tertiary hospital after obtaining approval from the Institutional Review Board (Internal Code 56/2017) and in accordance with the Declaration of Helsinki. Due to the retrospective nature of the research, we were exempted by the same Institutional Review Board of the Complejo Hospitalario Torrecárdenas from asking the patients for informed consent.

A sample size was not calculated due to the retrospective nature of the study. Therefore, the power of the study to detect as statistically significant the difference found between groups in the primary outcome variable was addressed in a *post hoc* analysis using GRANMO v7.11 program (Institut Municipal d’Investigació Mèdica, Barcelona, Spain).

### Target population

Adult patients referred to the Pain Clinic due to painful ulcers from January 2013 to December 2016 were eligible for this study. Patients suffering from nonvascular ulcers and patients not treated on an outpatient basis were excluded. All other patients were included for analysis.

### Comparators

Study groups were made up of patients who received usual analgesic treatment (CONVENTIONAL) and patients who received topical sevoflurane in addition to usual care (SEVOFLURANE).

### Time horizon

For CEAs focused on the healing of chronic wounds, long-term timeframes are usually chosen, frequently a one-year time horizon [22, 23], to account as much as possible for circumstances that could arise during the slow process of healing [22]. Thus, a one-year time horizon was also chosen for the present research, although it was focused on pain rather than healing.

### Data source

Clinical charts were reviewed to collect information on the patients’ demographics and the characteristics of their ulcers during the year of follow-up, including pain and analgesics consumption. Additionally, consumption of any other health resources, such as visits for consults or hospital admissions for any reason, were also collected.

### Study perspective

The perspective was that of the payer (public healthcare system); therefore, only direct medical costs were considered.

### Choice and measurement of health outcomes

Pain reduction was chosen as the primary health outcome for effectiveness. Sum of Pain Intensity Difference (SPID) was the indicator selected to summarize the evolution of pain scores from the first to the last visit during the year of follow-up. SPID was obtained as follows: pain scored at every visit (rated according to the Numerical Rating Scale [NRS]) was subtracted from the pain scored at the first visit to the Pain Clinic, and the result was multiplied by the fraction of time elapsed since every previous evaluation [25]. Hence, the higher the SPID, the greater the effectiveness.

Secondary indicators of health outcomes were leg amputation rate; patients’ mortality rate; ulcer healing rate; area of main ulcer at the 12^th^ month; absolute NRS-points of pain at the 12^th^ month; pain-reduction rate; and opioid consumption due to pain, expressed as oral morphine equivalents [26].

### Health resources consumed and related costs

The annual cost per patient was calculated by multiplying the health resources consumed (measured in natural units) by its specific unit costs. All costs were adjusted to € 2016 by using the Consumer Price Index [27]. Since the time horizon was one-year, no discount rate was applied.

For each patient, health resources collected were consumption of sevoflurane and other analgesics, and visits for consults and hospital admissions. All types of visits and hospitalizations were considered for the base-case analysis regardless of whether they could be directly related to the ulcers or not, to be sure not to miss costs derived from unexpected drug adverse effects or from any other unexpected complications related to the ulcers.

To calculate unit costs, three main health resources were taken into account: 1) sevoflurane consumption; 2) consumption of conventional analgesics and adjuvants; and 3) consumption of other health resources as outpatient consultations and hospital admissions.

1. To calculate unit costs related to sevoflurane consumption, three items were considered. First, the cost of a 250mL-bottle of sevoflurane, which was provided by the Pharmacy Department. Second, the cost of the 10 mL-syringes used, which was provided by the Administrative Department of the hospital. And third, the costs derived from the working time spent by a pharmacist to fill a 10 mL-syringe with sevoflurane; this item was estimated from health workers’ salaries [28–31] under the assumption that the pharmacist will spend one hour to charge 200 syringes, and the cost for one working hour would be of 2016 €41.
2. The unit costs for conventional analgesics and adjuvants were calculated from the Database of the Knowledge Health Bot PLUS [32].
3. The unit costs for outpatient consults and hospital admissions were obtained from the officially published public health prices [33].

The unit hospitalization costs related to the base case used were based on Spanish Medicare diagnosis-related groups (DRGs). For some diagnoses, DRGs consider two different costs depending on whether the patients suffered or not from complications while admitted; for such diagnoses, the specific cost with or without complications was attributed to each patient in a case-to-case basis after reviewing patients’ charts.

### Assumptions

For patients lacking a real pain score at the end of the follow-up period because they did not complete the period of follow-up because of death or leg amputation, the last available pain score was taken as the final pain score.

Costs derived from cleansing the ulcers at the Pain Clinic were not included for analysis because they were supposed to be a common expenditure for both groups and, besides, they were considered to be included in the costs derived from the visit itself.

### Cost-effectiveness analysis (CEA)

This CEA was not carried out in a frequentist approach but in a Bayesian framework, which allows for the incorporation of a priori information, if available, and to interpret the results in terms of probability [34–38]. To do this, guidelines published by the International Society for Pharmacoeconomics and Outcomes Research to improve the quality of health economic evaluations (Consolidated Health Economic Evaluation Reporting Standards; see S1 Table) [39, 40], retrospective studies [41] and real-world data studies [42] were followed. In addition, the STROBE guidelines were also followed [43].

Taking into consideration that the work was based on observational data, a selection bias could not be discarded. Thus, we planned to perform a linear regression analysis to ascertain if the main results could have been influenced by the assignation to the treatment groups. Specifically, a multiple regression analysis was conducted to identify the part of the difference in costs or effectiveness between groups that was not attributable to the treatment but instead to other covariates, specifically, baseline characteristics of the patients [44–51]. Therefore, covariates allow to reduce the bias and uncertainty of the estimation of the parameters, even if the treatment groups have similar characteristics [48–51]. The specification of this regression model was properly evaluated as it fitted the checklist developed to assess statistical methods for addressing selection bias in CEAs based on observational data [52].

The final model included the following six covariates: *AHT* (arterial hypertension, 0 = No, 1 = Yes); *UlcerDuration*, expressed as months; *UlcerNumber*, measured from 1 to 4 ulcers as this variable was truncated at 4 ulcers; *UlcerDepth*, dichotomized into superficial (0) and profound ulcers (1), being the epidermis and dermis the limit for superficial; *UlcerPain*, expressed as points of pain scored on the NRS, which ranges from 0 (no pain at all) to 10 (the worst imaginable pain); and *Treatment* (SEVOFLURANE = 1, CONVENTIONAL = 0). Details about variable selection are provided in the S2 File.

Thus, for the patient *i*, the linear regression model to explain *Costs* and *Effectiveness*, measured by *SPID*, were as follows:

$$Costs_{i}=\beta_{11}+\beta_{12}\times AHT_{i}+\beta_{13}\times UlcerDuration_{i}+\beta_{14}\times UlcerNumber_{i}+\beta_{15}\times UlcerDepth_{i}+\beta_{16}\times UlcerPain_{i}+\beta_{17}\times Treatment_{i}+\varepsilon_{1i}$$ (1)

$$Effectiveness_{i}=\beta_{21}+\beta_{22}\times AHT_{i}+\beta_{23}\times UlcerDuration_{i}+\beta_{24}\times UlcerNumber_{i}+\beta_{25}\times UlcerDepth_{i}+\beta_{26}\times UlcerPain_{i}+\beta_{27}\times Treatment_{i}+\varepsilon_{2i}$$ (2)

To conduct this Bayesian CEA, a multivariate normal distribution for effectiveness and log-transformed total costs was assumed; costs were log-transformed as they were asymmetrically distributed. Additionally, non-informative proper priors were considered, in particular a multivariate normal prior distribution with a mean vector of zeros and a covariance matrix 10^5^ x Identity-matrix(7) for the vector of means, and a Wishart distribution with 2 degrees of freedom and covariance matrix Identity-matrix(2) was considered as the prior distribution for the variance-covariance matrix.

The expected means for the *Costs* and *Effectiveness* and for all of the coefficients of the model were estimated from the posterior distributions, as well as their 95% Bayesian credible intervals. The estimation of the posterior distribution of the coefficients was performed using Markov Chain Monte Carlo (MCMC).[53, 54] A first burn-in sample with 10,000 simulations was calculated and then discarded. Further, 100,000 simulations were run from which the main statistics of the coefficients were calculated. CEA was conducted using OpenBUGS. The S3 File contains the OpenBUGS codes for our model.

Usually, β-coefficients for the covariate *Treatment* can be interpreted as the incremental cost (β_17_) in Eq (1) and the incremental effectiveness (β_27_) in Eq (2). However, this interpretation is invalid in the case where a variable was log-transformed, as it was the case for our variable *Costs*. Therefore, incremental cost cannot be considered as the value of the β-coefficient for *Treatment* (β_17_). Under these conditions, the costs ratio (costs of SEVOFLURANE divided by costs of CONVENTIONAL) is preferred over the incremental cost (costs of SEVOFLURANE minus costs of CONVENTIONAL). The costs ratio can be obtained from the exponential transformation of the β-coefficient associated with the covariate *Treatment*, namely, exp (β_17_). Thus, relative incremental cost attributed to sevoflurane treatment, the treatment under evaluation compared to usual care, can be expressed as (exp (β_17_) − 1) x 100.

If needed, the incremental cost-effectiveness ratio (ICER) would be calculated as the ratio between the incremental cost and the incremental effectiveness, expressed as euros per natural unit of effectiveness gained.

Finally, an x-y scatterplot was built to graphically show the posterior costs ratio and incremental effectiveness (the cost-effectiveness plane). Moreover, the probability of a preference for sevoflurane treatment was displayed as a function of the willingness to pay for increasing effectiveness in a natural unit (the cost-effectiveness acceptability curve).

### Sensitivity analyses

To determine the robustness of the model, three types of sensitivity analyses were conducted:

1. To conduct *one-way sensitivity analyses*, the costs of hospitalization was the variable selected because it had the potential for a high uncertainty and, besides, costs derived from hospitalization represented the highest percentage of the total costs. Therefore, base-case cost-effectiveness analysis (CEA) was repeated after calculating the costs derived from hospitalization in three different ways:
  - The hospitalization costs were based on the Spanish Medicare diagnosis-related groups (DRGs), as for the base-case CEA, yet the costs for each admission were calculated in two ways: first, considering that all patients had suffered from complications while admitted; second, considering that no patient suffered from such complications.
  - The hospitalization costs were based on the Spanish Medicare DRGs and each admission was assigned a cost with or without complications on a case-to-case basis, as for the base-case CEA, yet only admissions attributed to two specific conditions were considered: admissions attributed to the ulcers by any reason, or admissions attributed to the pain caused by the ulcers.
  - Instead of costs based on the Spanish Medicare DRGs, costs for each admission were calculated by multiplying the cost assigned to every day of hospitalization [33] by the length of stay.
2. Second, the base-case CEA was repeated after excluding the *subgroup of patients* who experienced a major negative outcome before completing a year of follow-up, namely, eight patients who died and three patients whose legs were amputated. Pain was the main clinical variable and, for those patients, pain from the event until the year of follow-up was not measured but extrapolated.
3. Lastly, *extreme scenario analyses* were conducted considering the worst scenario for SEVOFLURANE: values for the variable *Costs* were increased by 25% for SEVOFLURANE and reduced by 25% for CONVENTIONAL, whereas values for the variable *Effectiveness (*SPID*)* were reduced by 25% for SEVOFLURANE and increased by 25% for CONVENTIONAL. The concrete value of 25% was selected *post hoc* after rounding by an excess of the limits of the 95% confidence intervals found for *Costs* and *Effectiveness* (SPID*)*.

### Statistical analyses

Categorical variables are summarized as number and percentage; chi-square tests or Fisher’s exact tests were used to explore associations between variables.

Continuous variables are expressed as mean (SD). Student’s t-tests or Mann-Whitney U tests were used for comparisons according to the results of the Shapiro-Wilks normality test.

The level of significance was pre-established at 0.05. All of these exploratory statistical analyses were conducted using SPSS Statistics Version 24 (IBM SPSS Statistics, Version 22.0. Armonk, NY, USA). All relevant data are presented in S1 Dataset.

## Results

Seventy-five patients were referred to the Pain Clinic due to painful wounds during the study period. Nine of them were excluded because they suffered from painful wounds that were not to leg ulcers. Two patients suffering from painful leg ulcers were excluded because they were not treated on an outpatient basis.

Overall, 64 patients were included for analysis. The SEVOFLURANE group was composed of 38 patients treated with domiciliary topical sevoflurane in addition to usual care, while the CONVENTIONAL group was composed of 26 patients treated only with usual care.

### Demographic characteristics

Baseline characteristics of patients, leg ulcers, and pain were very similar between groups (Table 1).

**Table 1:** Baseline characteristics.

	SEVOFLURANE (n = 38)	CONVENTIONAL (n = 26)	P-value
**PATIENTS**			
Age (y)	70.4 (12.2)	71.9 (11.2)	0.681^a^
Sex, Female (%)	63	54	0.456^b^
Weight (kg)	69.5 (13.2)	71.85 (18.3)	0.869^a^
Diabetes mellitus (%)	63	81	0.130^b^
Arterial hypertension (%)	71	77	0.602^b^
**LEG ULCERS**			
Etiology, ischemic component (%)	92	96	0.640^c^
Duration (months)	24.8 (32.2)	16.2 (14.5)	0.864^a^
Ulcers per patient (n)	2.4 (2.3)	2.8 (2.6)	0.364^a^
Depth beyond dermis (%)	26	31	0.697^b^
Area, main ulcer (cm^2^)	9.2 (11.3)	10.1 (9.6)	0.430^a^
**ULCER-RELATED PAIN**			
Pain (NRS)	6.7 (1.6)	6.9 (1.4)	0.797^a^
Neuropathic pain (%)	53	62	0.481^b^
Daily analgesics, active principles (n)	3.0 (1.2)	3.0 (1.0)	0.732^a^
Patients taking any opioid (%)	87	85	1.000^c^
OME only for patients taking opioids (mg/d)	118.1 (74.6)	123.1 (89.1)	0.836^a^
	(n = 33)	(n = 22)	

Quantitative data are expressed as the mean (SD). NRS, Numerical Rating Scale; OME, Oral Morphine Equivalent.

^a^Mann-Whitney U test.

^b^Chi square test.

^c^Fisher exact test.

### Health outcomes

SEVOFLURANE patients showed a nonsignificant higher percentage of ulcer healing and a significant reduction in ulcer size.

Concerning pain, the SEVOFLURANE group presented significantly better results for all indicators of clinical effectiveness (Table 2).

**Table 2:** Health outcomes for the follow-up period (12 months).

	SEVOFLURANE (n = 38)	CONVENTIONAL (n = 26)	P-value
**PATIENTS**			
Patients admitted for any reason (%)	50	85	0.007^b^
Patients whose admissions were attributed to the ulcers (%)	21	54	0.009^b^
Patients whose admissions were attributed to ulcer pain (%)	8	38	0.004^b^
Leg amputation rate (%)	5	4	1.000^c^
Mortality rate (%)	11	15	0.705^c^
**LEG ULCERS**			
Healing rate (%)	40	27	0.299^b^
Area, main ulcer (cm^2^)	5.3 (12.2)	8.1 (10.2)	0.018^a^
**ULCER-RELATED PAIN**			
Pain (NRS)	1.1 (1.0)	4.1 (2.4)	<0.001^a^
Pain reduction rate (%)	84.1 (14.3)	39.1 (35.5)	<0.001^a^
Patients taking any opioids (%)	24	50	0.036^b^
OME only for patients taking opioids (mg/d)	51.9 (44.8)	144.5 (65.7)	0.001^a^
	(n = 9)	(n = 13)	
SPID	41.5 (18.1)	13.1 (10.4)	<0.001^a^

Quantitative data are expressed as the mean (SD). NRS, Numerical Rating Scale; OME, Oral Morphine Equivalent; SPID, Sum of Pain Intensity Difference.

^a^Mann-Whitney U test.

^b^Chi square test.

^c^Fisher exact test.

Accepting an alpha risk of 0.05 in a two sided test and using the widest standard deviation found in the SEVOFLURANE group as the common standard deviation for both groups (18.1), the power of the study was 100% to recognize as statistically significant the striking difference in SPID between groups.

### Costs analysis

Costs of analgesic drugs were significantly higher for the SEVOFLURANE group due to sevoflurane consumption, but the total costs of health resources consumed were significantly lower for SEVOFLURANE because patients in this group consumed significantly fewer health resources (Table 3).

**Table 3:** Health resources with their unit costs and associated total health costs.

	HEALTH RESOURCES (n)			UNIT COSTS (2016 €)	COSTS (€)		
	SEVOFLURANE (n = 38)	CONVENTIONAL (n = 26)	P-value		SEVOFLURANE (n = 38)	CONVENTIONAL (n = 26)	P-value
**Sevoflurane (mL)**	8784.0 (14116.5)	---	---	0.30	3240 (5657)	---	---
**10 mL-syringes**	878.8 (1411.7)	---	---	0.17	150 (242)	---	---
**Charging the syringes**	878.8 (1411.7)	---	---	0.21^a^	181 (293)	---	---
**COSTS OF SEVOFLURANE TREATMENT**	---	---	---	---	**3572 (6190)**	---	---
**Opioids**	*	*	---	*	382 (421)	564 (577)	0.216
**Nonopioids**	*	*	---	*	139 (155)	290 (325)	0.005
**COSTS OF ALL ANALGESICS, INCLUDING SEVOFLURANE**	**---**	**---**	---	---	**4093 (6224)**	**854 (680)**	<0.001
**PRIMARY CARE CONSULTATION, SCHEDULED**							
**-Medical**							
First visit	1.0 (0.0)	1.0 (0.0)	1.000	44	44 (0.2)	44 (0.2)	1.000
Next visits	2.6 (2.8)	5.0 (2.3)	<0.001	18	47 (50)	89 (41)	<0.001
**-Medical with nurse**							
First visit	0.4 (0.5)	0.8 (0.4)	<0.001	52	19 (25)	41 (21)	<0.001
Next visits	1.6 (4.3)	3.1 (3.7)	0.001	23	36 (99)	71 (83)	0.001
**-Nurse**	6.1 (5.9)	10.1 (7.2)	0.004	21	129 (124)	212 (150)	0.004
**PRIMARY CARE CONSULTATION, NONSCHEDULED**							
Simple	1.2 (1.7)	2.9 (2.1)	<0.001	49	58 (83)	144 (104)	<0.001
Discharge after observation	0.3 (0.8)	0.7 (1.3)	0.078	86	23 (72)	60 (109)	0.078
Referral to the hospital	0.5 (0.9)	2.0 (2.9)	0.003	111	56 (99)	218 (328)	0.003
**HOSPITAL CONSULTATION, SCHEDULED**							
First visit	1.0 (0.0)	1.0 (0.0)	1.000	114	115 (0.6)	114 (0.5)	1.000
Next visits	8.4 (8.9)	11.7 (11.2)	0.003	55	462 (485)	638 (618)	0.003
**HOSPITAL CONSULTATION, NONSCHEDULED**							
Discharge after consultation	0.8 (1.5)	1.5 (1.3)	0.009	144	114 (213)	211 (193)	0.009
With further admission	1.1 (1.9)	1.88 (1.4)	0.005	392	435 (759)	741 (549)	0.005
**HOSPITAL ADMISSIONS, DRGS**	1.13 (1.7)	2.00 (1.3)	0.004	Variable^b^	6900 (10893)	12692 (9030)	0.004
**HYPERBARIC CHAMBER**	2.6 (11.7)	1.6 (8.3)	0.744	65	180 (784)	113 (578)	0.794
**COSTS EXCLUDING ANALGESICS**	---	---	---	---	**8618 (12220)**	**15388 (9860)**	**0.001**
**TOTAL COSTS**	---	---	---	---	**12701 (15911)**	**16245 (10012)**	**0.038**

DRGs, Diagnosis-Related Groups. Data are expressed as mean (SD). Mann-Whitney U test for all comparisons.

*See S2 Table for details.

^a^ Costs attributed to a pharmacist to charge one single syringe.

^b^ Hospitalization costs were based on Spanish Medicare DRGs [33].

### Cost-effectiveness analysis

The Bayesian cost-effectiveness analysis for the base-case showed lower costs and higher effectiveness (SPID) for the SEVOFLURANE group (Table 4); consequently, this group was dominant over the CONVENTIONAL group.

**Table 4:** Statistical summary of costs and effectiveness (100,000 simulations MCMC).

	SEVOFLURANE		CONVENTIONAL		Incremental difference	
	Mean (SD)	95% CrI	Mean (SD)	95% CrI	Mean (SD)	95% CrI
**Costs (€)**	10250 (2194)	(7316; 14230)	19750 (4915)	(13260; 28750)	0.54 (0.15)	(**0.33; 0.83**)
**Effectiveness (SPID)**	41.37 (2.32)	(37.56; 45.16)	13.22 (2.82)	(8.59; 17.86)	28.15 (3.70)	**(22.06; 34.22)**

CrI not including the zero value are highlighted in bold. CrI, Credible Interval; MCMC, Markov Chain Monte Carlo; SPID, Sum of Pain Intensity Difference.

Regarding costs, *ceteris paribus*, SEVOFLURANE was associated with a reduction of 46% in costs compared to CONVENTIONAL, as indicated by the mean value of the costs ratio (0.54±0.15) with a posterior 95% Bayesian credible interval of 0.33 to 0.83 (Table 4).

Regarding effectiveness, *ceteris paribus*, patients in the SEVOFLURANE group experienced an increase of 28.15±3.70 natural units of effectiveness over the CONVENTIONAL, as indicated by the value of the β_27-_coefficient, which usually allows for estimating the incremental effectiveness; moreover, this increment was relevant since its posterior 95% Bayesian credible interval was 19.52 to 31.31 (Table 4).

Concerning costs in the regression model, the covariates *UlcerDuration*, *UlcerNumber*, *UlcerDepth*, and *Treatment* showed relevant explanatory power over *Costs* (Table 5). Specifically, the longer the duration of the ulcer, the greater the number of ulcers, and the deeper the ulcers, the higher the costs. In contrast, total costs were lower for patients treated with topical sevoflurane.

**Table 5:** Estimations of the posterior distribution of the β-coefficients for the cost-effectiveness analysis (100,000 simulations MCMC).

		Mean (SD)	95% CrI
**Costs**	β_11_ *intercept*	7.65 (0.73)	**(6.44; 8.85)**
	β_12_ *AHT*	0.43 (0.31)	(-0.09; 0.94)
	β_13_ *UlcerDuration*	0.02 (0.01)	**(0.01; 0.03)**
	β_14_ *UlcerNumber*	0.30 (0.11)	**(0.12; 0.47)**
	β_15_ *UlcerDepth*	0.96 (0.32)	**(0.42; 1.49)**
	β_16_ *UlcerPain*	0.01 (0.09)	(-0.14; 0.17)
	β_17_ *Treatment*	-0.65 (0.28)	**(-1.10; -0.19**)
	Costs ratio (exp β_17_)	0.54 (0.15)	(**0.33; 0.83**)
**Effectiveness**	β_21_ *intercept*	-22.44 (9.79)	**(-38.39; -6.32)**
	β_22_ *AHT*	1.38 (4.19)	(-5.51; 8.25)
	β_23_ *UlcerDuration*	0.12 (0.07)	**(0.00; 0.23)**
	β_24_ *UlcerNumber*	0.60 (1.42)	(-1.74; 2.95)
	β_25_ *UlcerDepth*	1.33 (4.33)	(-5.78; 8.43)
	β_26_ *UlcerPain*	4.51 (1.26)	**(2.44; 6.58)**
	β_27_ *Treatment*	28.15 (3.70)	**(22.06; 34.22)**

CrI not including the zero value are highlighted in bold. AHT, Arterial Hypertension; CrI, Credible Interval; MCMC:,Markov Chain Monte Carlo.

Concerning effectiveness in the regression model, the covariates showing relevant explanatory power over *Effectiveness* were *UlcerDuration*, *UlcerPain* and *Treatment* (Table 5). Specifically, the longer the duration of the ulcer and the higher the baseline pain, the higher the SPID, which implies more pain reduction; but the most remarkable pain reduction was found for patients who had been treated with sevoflurane (ß_27_ = 28.15).

**Fig 1:**
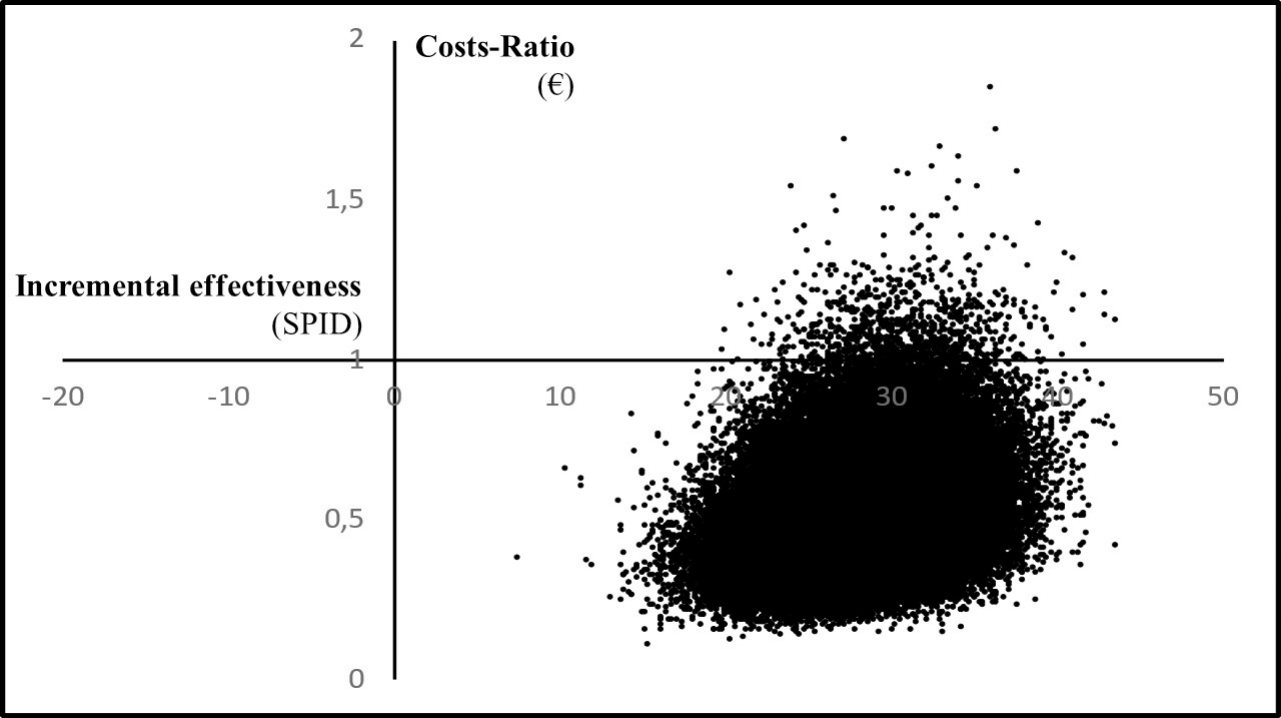
Cost-effectiveness plane.

**Fig 2:**
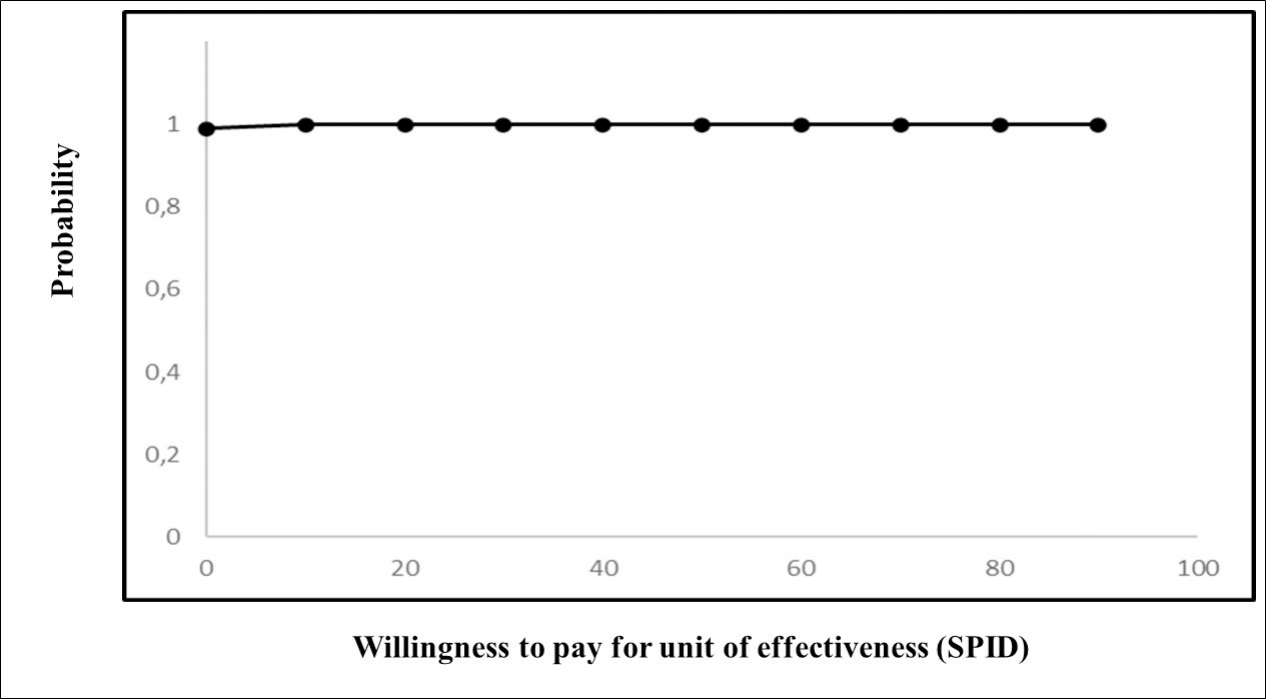
Cost-effectiveness acceptability curve.

The Bayesian probability for SEVOFLURANE being less expensive was found to be as high as 99%, whereas the probability for SEVOFLURANE being more effective in reducing pain was 100%. Overall, the estimated probability for SEVOFLURANE being dominant was noticeably high (99%). Graphically, the preference for sevoflurane treatment over usual care is shown in the cost-effectiveness plane (Fig 1) and in the cost-effectiveness acceptability curve (Fig 2).

Scatterplot showing the posterior costs ratio and incremental effectiveness measured by the natural unit of effectiveness. SPID, Sum of Pain Intensity Difference.

The curve shows the cost-effectiveness probabilities for SEVOFLURANE by different degrees of willingness to pay for the natural unit of effectiveness. SPID, Sum of Pain Intensity Difference.

### Sensitivity analysis

First, SEVOFLURANE was found to be the dominant alternative in all one-way sensitivity analyses conducted; noteworthy, the probability for SEVOFLURANE being dominant was always higher than 80%.

After excluding eight patients who died and three patients who suffered from leg amputation during the year of follow-up, the final subgroups for the second type of sensitivity analyses were made up of 32 patients for SEVOFLURANE and 21 patients for CONVENTIONAL. After conducting these new analyses, SEVOFLURANE was also found to be the dominant alternative, with an estimated probability of 98%.

Lastly, the lowest probability for SEVOFLURANE to be dominant was found for the worst scenario for sevoflurane, but even so, its probability was as high as 69% (S3 and S4 Tables).

## Discussion

This study was conducted on patients suffering from painful nonrevascularizable chronic leg ulcers. Compared to usual analgesic care alone, the addition of topical sevoflurane was the dominant alternative since it was associated with both a reduction in costs and an improvement in analgesic effect, making it cost-effective. After further analysis, the probability for sevoflurane being the dominant alternative was found to be as high as 99%. Such a remarkable result seemed to not have been influenced by an eventual selection bias when allocating patients, as it will be further discussed in the Limitations section.

Economic evaluations focused on topical sevoflurane were lacking until now, precluding our results for direct comparison. That is logical, considering that this new indication was first reported only 10 years ago [9].

Regarding effectiveness, the probability of SEVOFLURANE being more effective was 100% for the base-case and for all sensitivity analyses, even under the worst scenario for this group. This finding strongly agrees with a growing body of literature on the great clinical effectiveness of topical sevoflurane for painful vascular ulcers and other wounds of different etiologies [9–21]. In a recent retrospective study with a similar design, intensive care patients suffering from painful pressure ulcers and experiencing adverse effects caused by opioids were prescribed topical sevoflurane; compared to opioids alone, the addition of sevoflurane yielded results in effectiveness similar to ours, as pain scores were significantly lower and opioid consumption was significantly reduced [21].

The addition of sevoflurane increased the costs of analgesic medication despite reducing the consumption of conventional analgesics, as they are usually cheaper than sevoflurane. However, total costs were lower for the SEVOFLURANE group due to remarkably lower consumption of more expensive health resources, namely, visits for consults and hospital admittances. Interestingly, these patients were less frequently admitted for ulcer pain or for any other reason related to their ulcers. Whether the reduction in conventional analgesics consumption was followed by less consumption of health resources due to a further reduction of unwanted events could not be ascertained due to the retrospective nature of the study, but this hypothesis is attractive and deserves further research.

The CONVENTIONAL group was made up of patients referred to the Pain Clinic who did not benefit from sevoflurane due to safety concerns. This problem could be easily solved by treating them at the primary care center [17] or even at home by a domiciliary support team [18]. In our opinion, both strategies would be cost-effective, since the costs arising from the visits would be counterbalanced by the clinical benefits achieved.

### Study perspective

As many other authors did [22, 23], we chose a public payer perspective instead of a societal one. Therefore, indirect costs related to the societal perspective, such as those derived from loss of productivity, were excluded from the analysis. In our opinion, a public payer perspective is suitable for several reasons. First, indirect costs may have a little impact on total costs; in a study conducted in Germany describing costs associated with chronic leg ulcers, indirect costs represented only 10% of the total costs [55]. Second, patients suffering from chronic leg ulcers are usually retired because frequently they are elderly, as our patients were; thus, costs derived from loss of productiveness were not applicable [56]. Not surprisingly, the societal perspective has been scarcely applied so far; for instance, the societal perspective was reported in only 14% of CEAs conducted on chronic wounds [22].

As the chosen perspective was that of the public payer, only directs costs were considered for the CEA. We aimed not to miss any cost derived from the consumption of health resources because of the ulcers, and it was a difficult task to properly discriminate which costs were related or unrelated to the ulcers; for this reason, we decided to consider for the base-case analysis all costs derived from all resources collected from the charts. Nevertheless, in order to conduct two sensitivity analyses, costs were reassigned either as related to the ulcers by any reason or related exclusively to the pain caused by the ulcers; the estimated probability for SEVOFLURANE to be dominant was also high after both re-analyses, specifically 90% and 81%, respectively.

### Strengths

In our opinion, this work has several strengths that deserve comment.

First, CEAs in the field of chronic wounds have been mainly focused on several aspects of healing, while pain has been scarcely studied so far. To the best of our knowledge, this is the first time a CEA conducted in the setting of chronic wounds focused on pain, and our encouraging results strongly suggest that this symptom should be more frequently taken into account in future research.

Second, this was not a clinical trial but an observational study. As such, patients were treated by their attending physician according to the best clinical judgement. Thus, our results are based on real-world data, which allows for generalization of the results to the day-to-day practice [57, 58]. In our opinion, this approach represents an advantage over works that try to model the natural history of the disease based on population data [59].

### Limitations

In addition to the inherent limitations common to all CEAs (see CHEERS checklist in S1 Table in supporting information), the present study has the following particular limitations.

The main limitation of this retrospective observational study is that a selection bias could not be completely ruled out. Safety concerns were the main factor in deciding whether any specific patient was suitable to be treated with sevoflurane. Nonetheless, both groups showed quite similar baseline characteristics, whereas their pain evolution was markedly different from the very beginning. Even so, to try to eliminate any eventual bias and to reduce uncertainty, we conducted a regression model considering those variables that could affect cost and effectiveness to obtain the true effect of the treatment on the CEA result. In addition to *Treatment*, some other variables such as *UlcerDuration*, *UlcerDepth*, and *UlcerPain* showed relevant explanatory power on costs or effectiveness. However, costs were barely influenced by any of these variables, whereas effectiveness was markedly influenced only by *Treatment*, as indicated by the values of the ß-coefficients (Table 5). Considering this, we do not believe our results were influenced by selection bias, even when accepting that such bias could have existed.

Second, the sample size was relatively small—although quite similar to many other studies on chronic wounds—[22] because of two main reasons. We conducted a Spanish single center study because, currently, there is no other Pain Clinic treating patients with domiciliary topical sevoflurane, precluding the possibility of a multicenter study. The sample size was further limited by the fact that, according to the approved protocol, only patients suffering from nonrevascularizable leg ulcers were suitable to be referred to the Pain Clinic for palliative analgesic treatment. Nevertheless, we found *post hoc* that our study was not underpowered.

Third, this specificity in design could affect the generalization of our findings to other settings or other types of wounds. Generalization to other countries should also be done with caution since unit costs could be different in other socio-economic contexts. However, the present CEA showed robust results favoring SEVOFLURANE, which was mainly due to the remarkable difference in the analgesic effectiveness of topical sevoflurane. Of note, SEVOFLURANE remained dominant even under extreme scenario analysis, which was the worst scenario for this group. Therefore, SEVOFLURANE can be considered dominant to CONVENTIONAL in terms of cost-effectiveness with a high degree of confidence [60].

## Conclusions

In conclusion, the results of this retrospective observational cohort study strongly suggest that the addition of domiciliary topical sevoflurane to the usual care provided by a Pain Clinic for nonrevascularizable painful leg ulcers is a cost-effective alternative compared to usual care alone. The addition of sevoflurane was followed by a reduction in costs and, of note, by a great improvement in analgesic effectiveness. Therefore, healthcare decision-makers should consider this new alternative, as it could lead to a more efficient application of healthcare resources.

## Supporting information

S1 File Background on the use of topical sevoflurane. (PDF)

S2 File Selection of covariates for Bayesian regression model. (PDF)

S3 File OpenBUGS codes for cost-effectiveness analysis. (PDF)

S1 Table CHEERS checklist. (PDF)

S2 Table Mean consumption of pharmaceutical formulations for each group. (PDF)

S3 Table Sensitivity analyses: Statistical summary of costs and effectiveness (100,000 simulations MCMC). (PDF)

S4 Table Sensitivity analyses: Estimations of the posterior distribution of the β-coefficients and of the probabilities related to the cost-effectiveness analysis (100,000 simulations MCMC). (PDF)

S1 Dataset (XLSX)

S1 Striking image (TIF)

## References

[1] Martinengo L, Olsson M, Bajpai R, Soljak M, Upton Z, Schmidtchen A, et al. Prevalence of chronic wounds in the general population: systematic review and meta-analysis of observational studies. Ann Epidemiol. 2019;29: 8–15. doi: 10.1016/j.annepidem.2018.10.005 30497932

[2] Chan B, Cadarette S, Wodchis W, Wong J, Mittmann N, Krahn M. Cost-of-illness studies in chronic ulcers: a systematic review. J Wound Care. 2017;26: S4–S14. doi: 10.12968/jowc.2017.26.Sup4.S4 28379102

[3] Olsson M, Järbrink K, Divakar U, Bajpai R, Upton Z, Schmidtchen A, et al. The humanistic and economic burden of chronic wounds: A systematic review. Wound Repair Regen. 2019;27: 114–125. doi: 10.1111/wrr.12683 30362646

[4] Nussbaum SR, Carter MJ, Fife CE, DaVanzo J, Haught R, Nusgart M, et al. An economic evaluation of the impact, cost, and medicare policy implications of chronic nonhealing wounds. Value Health. 2018;21: 27–32. doi: 10.1016/j.jval.2017.07.007 29304937

[5] Green J, Jester R, McKinley R, Pooler A. The impact of chronic venous leg ulcers: a systematic review. J Wound Care. 2014;23: 601–612. doi: 10.12968/jowc.2014.23.12.601 25492276

[6] Laoire ÁN, Murtagh FEM. Systematic review of pharmacological therapies for the management of ischaemic pain in patients with non-reconstructable critical limb ischaemia. BMJ Support Palliat Care. 2018;8: 400–410. doi: 10.1136/bmjspcare-2017-001359 28835456 PMC6287571

[7] Pérez Lázaro JJ, Fernández Ruiz I, Tejedor Fernández M, Guerra de Hoyos JA, Jiménez Rodríguez M, de Pazzis Die de Ortega M, et al. Identifying and prioritising adverse episodes and failures related to patient safety in Pain Treatment Units [in Spanish]. Rev Esp Anestesiol Reanim. 2012;59: 423–429. doi: 10.1016/j.redar.2012.04.023 22742871

[8] Argoff CE. Topical Analgesics in the Management of Acute and Chronic Pain. Mayo Clin Proc. 2013;88: 195–205. doi: 10.1016/j.mayocp.2012.11.015 23374622

[9] Gerónimo-Pardo M, Martínez-Monsalve A, Martínez-Serrano M. Analgesic effect of topical sevoflurane on venous leg ulcer with intractable pain. Phlebologie. 2011;40: 95–97. doi: 10.1055/s-0037-1621757

[10] Martínez-Monsalve A, Gerónimo-Pardo M. Analgesic effect of topical sevoflurane on ischaemic wound in a patient with ischaemic cardiopathy and respiratory failure secondary to morphine [in Spanish]. Heridas y Cicatrización. 2011;6: 46–49.

[11] Martínez-Monsalve A, Gerónimo-Pardo M. The analgesic effect of topical sevoflurane on painful varicose ulcers in ambulatory patients [in Spanish]. Heridas y Cicatrización. 2013;1: 16–19.

[12] Dámaso Fernández-Ginés F, Cortiñas-Sáenz M, Mateo-Carrasco H, de Aranda AN-G, Navarro-Muñoz E, Rodríguez-Carmona R, et al. Efficacy and safety of topical sevoflurane in the treatment of chronic skin ulcers. Am J Heal Pharm. 2017;74: e176–e182. doi: 10.2146/ajhp151008 28438822

[13] Imbernon-Moya A, Ortiz de Frutos FJ, Sanjuan-Alvarez M, Portero-Sánchez I. Pain, quality of life, and functional capacity with topical sevoflurane application for chronic venous ulcers: a retrospective clinical study. EJVES Short Reports. 2017;36: 9–12. doi: 10.1016/j.ejvssr.2017.08.001 29296683 PMC5626915

[14] Amores Valenciano P, Navarro Castillo A, Romero Cebrián A, Gerónimo-Pardo M. Topical sevoflurane for rescue analgesia in refractory pain due to chronic venous ulcers [in Spanish]. Emergencias. 2018;30: 138. 29547243

[15] Martínez-Monsalve A, Selva-Sevilla C, Gerónimo-Pardo M. Analgesic effectiveness of topical sevoflurane to perform sharp debridement of painful wounds. J Vasc Surg. 2019;69: 1532–1537. doi: 10.1016/j.jvs.2018.08.175 30612826

[16] Fernández-Ginés FD, Cortiñas-Sáenz M, Selva-Sevilla C, Gerónimo-Pardo M. Sevoflurane topical analgesia for intractable pain with suicidal ideation. BMJ Support Palliat Care. 2020; bmjspcare-2019-002023. doi: 10.1136/bmjspcare-2019-002023 32601149

[17] Pinar Sánchez J, Gerónimo-Pardo M. Topical sevoflurane as rescue therapy in a frail patient suffering from leg ulcers causing refractory pain and superinfected by methicillin-resistant Staphylococcus aureus and sensitive Pseudomonas aeruginosa. A case report [in Spanish]. Med Paliativa. 2020;27: 141–145. doi: 10.20986/medpal.2020.1130/2019

[18] Navarro-Buendía GA, Satorres-Pérez M, Jiménez-Ruíz L. Efects of topical sevoflurane in the treatment of vascular ulcers. Report of a clinical case [in Spanish]. Hosp a Domic. 2020;4: 45–50. doi: 10.22585/hospdomic.v4i2.101

[19] Padilla del Rey M, Gerónimo-Pardo M, García Fernández M, Cartagena Sevilla J. Painful diabetic foot ulcer amputation avoided thanks to topical sevoflurane [in Spanish]. Rev Soc Esp Dolor. 2019;26: 253.

[20] Fernández-Ginés FD, Cortiñas-Sáenz M, Navajas-Gómez de Aranda A, Navas-Martinez MDC, Morales-Molina JA, Sierra-García F, et al. Palliative analgesia with topical sevoflurane in cancer-related skin ulcers: a case report. Eur J Hosp Pharm. 2019;26: 229–232. doi: 10.1136/ejhpharm-2017-001421 31338175 PMC6613928

[21] Fernández‐Ginés FD, Cortiñas‐Sáenz M, Agudo‐Ponce D, Navajas‐Gómez de Aranda A, Morales‐Molina JA, Fernández‐Sánchez C, et al. Pain reduction of topical sevoflurane vs intravenous opioids in pressure ulcers. Int Wound J. 2020;17: 83–90. doi: 10.1111/iwj.13235 31762163 PMC7948686

[22] Tricco AC, Cogo E, Isaranuwatchai W, Khan PA, Sanmugalingham G, Antony J, et al. A systematic review of cost-effectiveness analyses of complex wound interventions reveals optimal treatments for specific wound types. BMC Med. 2015;13: 90. doi: 10.1186/s12916-015-0326-3 25899057 PMC4405871

[23] Carter MJ. Economic evaluations of guideline-based or strategic interventions for the prevention or treatment of chronic wounds. Appl Health Econ Health Policy. 2014;12: 373–389. doi: 10.1007/s40258-014-0094-9 24615160 PMC4110411

[24] Gordon L, Edwards H, Courtney M, Finlayson K, Shuter P, Lindsay E. A cost-effectiveness analysis of two community models of care for patients with venous leg ulcers. J Wound Care. 2006;15: 348–353. doi: 10.12968/jowc.2006.15.8.26942 17001943

[25] D’Haese Vanlersberghe, Umbrain Camu. Pharmaco-economic evaluation of a disposable patient-controlled analgesia device and intramuscular analgesia in surgical patients. Eur J Anaesthesiol. 1998;15: 297–303. doi: 10.1046/j.1365-2346.1998.00292.x 9649988

[26] Nielsen S, Degenhardt L, Hoban B, Gisev N. A synthesis of oral morphine equivalents (OME) for opioid utilisation studies. Pharmacoepidemiol Drug Saf. 2016;25: 733–737. doi: 10.1002/pds.3945 26693665

[27] Instituto Nacional de Estadística. [cited 2 Jun 2019]. Available: https://www.ine.es/jaxiT3/Tabla.htm?t=22350

[28] Servicio Andaluz de Salud: Retribuciones del personal de Centros e Instituciones Sanitarias. Ejercicio 2012. [cited 2 Jun 2019]. Available: https://www.sspa.juntadeandalucia.es/servicioandaluzdesalud/sites/default/files/sincfiles/wsas-media-sas_normativa_mediafile/2019/Resolución00022012.pdf

[29] Servicio Andaluz de Salud: Retribuciones del personal de Centros e Instituciones Sanitarias. Ejercicio 2015. [cited 2 Jun 2019]. Available: https://www.sspa.juntadeandalucia.es/servicioandaluzdesalud/sites/default/files/sincfiles/wsas-media-sas_normativa_mediafile/2019/ResolucionSSA_00182015.pdf

[30] Servicio Andaluz de Salud: Retribuciones del personal de Centros e Instituciones Sanitarias. Ejercicio 2016. [cited 2 Jun 2019]. Available: https://www.sspa.juntadeandalucia.es/servicioandaluzdesalud/sites/default/files/sincfiles/wsas-media-sas_normativa_mediafile/2019/20160418_Resolucion_0136_2016.pdf

[31] Servicio Andaluz de Salud: Retribuciones del personal de Centros e Instituciones Sanitarias. Ejercicio 2017. [cited 2 Jun 2019]. Available: https://www.sspa.juntadeandalucia.es/servicioandaluzdesalud/sites/default/files/sincfiles/wsas-media-sas_normativa_mediafile/2019/R_0193_2017.pdf

[32] Knowledge Health Bot PLUS. [cited 2 Jun 2019]. Available: https://botplusweb.portalfarma.com/

[33] Boletín Oficial de la Junta de Andalucía. [cited 1 Dec 2019]. Available: https://www.juntadeandalucia.es/boja/2005/210/28

[34] Briggs AH. A Bayesian approach to stochastic cost-effectiveness analysis. Health Econ. 1999;8: 257–261. doi: 10.1002/(sici)1099-1050(199905)8:3&lt;257::aid-hec427&gt;3.0.co;2-e 10348420

[35] Heitjan DF, Moskowitz AJ, Whang W. Bayesian estimation of cost-effectiveness ratios from clinical trials. Health Econ. 1999;8: 191–201. doi: 10.1002/(sici)1099-1050(199905)8:3&lt;191::aid-hec409&gt;3.0.co;2-r 10348414

[36] O’Hagan A, Stevens JW, Montmartin J. Bayesian cost-effectiveness analysis from clinical trial data. Stat Med. 2001;20: 733–753. doi: 10.1002/sim.861 11241573

[37] O’Hagan A, Stevens JW. The probability of cost-effectiveness. BMC Med Res Methodol. 2002;2: 5. doi: 10.1186/1471-2288-2-5 11914138 PMC100784

[38] O’Hagan A, Stevens JW. A framework for cost-effectiveness analysis from clinical trial data. Health Econ. 2001;10: 303–315. doi: 10.1002/hec.617 11400253

[39] McGhan WF, Al M, Doshi JA, Kamae I, Marx SE, Rindress D. The ISPOR good practices for quality improvement of cost-effectiveness research Task Force Report. Value Health. 2009;12: 1086–1099. doi: 10.1111/j.1524-4733.2009.00605.x 19744291

[40] Husereau D, Drummond M, Petrou S, Carswell C, Moher D, Greenberg D, et al. Consolidated Health Economic Evaluation Reporting Standards (CHEERS)—explanation and elaboration: A report of the ISPOR Health Economic Evaluation Publication Guidelines Good Reporting Practices Task Force. Value Health. 2013;16: 231–250. doi: 10.1016/j.jval.2013.02.002 23538175

[41] Berger ML, Mamdani M, Atkins D, Johnson ML. Good research practices for comparative effectiveness research: defining, reporting and interpreting nonrandomized studies of treatment effects using secondary data sources: the ISPOR good research practices for retrospective database analysis task force. Value Health. 2009;12: 1044–1052. doi: 10.1111/j.1524-4733.2009.00600.x 19793072

[42] Berger ML, Sox H, Willke R, Brixner D, Eichler H-G, Goettsch W, et al. Good practices for real-world data studies of treatment and/or comparative effectiveness: Recommendations from the joint ISPOR-ISPE Special Task Force on real-world evidence in health care decision making. Value Health. 2017;20: 1003–1008. doi: 10.1016/j.jval.2017.08.3019 28964430

[43] von Elm E, Altman DG, Egger M, Pocock SJ, Gøtzsche PC, Vandenbroucke JP. The strengthening the reporting of observational studies in epidemiology (STROBE) statement: guidelines for reporting observational studies. J Clin Epidemiol. 2008;61: 344–349. doi: 10.1016/j.jclinepi.2007.11.008 18313558

[44] Hoch JS, Briggs AH, Willan AR. Something old, something new, something borrowed, something blue: a framework for the marriage of health econometrics and cost-effectiveness analysis. Health Econ. 2002;11: 415–430. doi: 10.1002/hec.678 12112491

[45] Willan AR, Briggs AH, Hoch JS. Regression methods for covariate adjustment and subgroup analysis for non-censored cost-effectiveness data. Health Econ. 2004;13: 461–475. doi: 10.1002/hec.843 15127426

[46] Nixon RM, Thompson SG. Methods for incorporating covariate adjustment, subgroup analysis and between-centre differences into cost-effectiveness evaluations. Health Econ. 2005;14: 1217–1229. doi: 10.1002/hec.1008 15945043

[47] Manca A, Rice N, Sculpher MJ, Briggs AH. Assessing generalisability by location in trial-based cost-effectiveness analysis: the use of multilevel models. Health Econ. 2005;14: 471–485. doi: 10.1002/hec.914 15386662

[48] Vázquez-Polo FJ, Negrín MA, Badia X, Roset M. Bayesian regression models for cost-effectiveness analysis. Eur J Health Econ. 2005;6: 45–52. doi: 10.1007/s10198-004-0256-z 15517461

[49] Vázquez-Polo FJ, Negrín MA, González López-Valcárcel B. Using covariates to reduce uncertainty in the economic evaluation of clinical trial data. Health Econ. 2005;14: 545–557. doi: 10.1002/hec.947 15497202

[50] Negrín MA, Vázquez-Polo FJ. Incorporating model uncertainty in cost-effectiveness analysis: A Bayesian model averaging approach. J Health Econ. 2008;27: 1250–1259. doi: 10.1016/j.jhealeco.2008.03.005 18490067

[51] Negrín MA, Vázquez-Polo FJ, Martel M, Moreno E, Girón FJ. Bayesian variable selection in cost-effectiveness analysis. Int J Environ Res Public Health. 2010;7: 1577–1596. doi: 10.3390/ijerph7041577 20617047 PMC2872346

[52] Kreif N, Grieve R, Sadique MZ. Statistical methods for cost-effectiveness analyses that use observational data: A critical appraisal tool and review of current practice. Health Econ (United Kingdom). 2013;22: 486–500. doi: 10.1002/hec.2806 22447531

[53] Geman S, Geman D. Stochastic relaxation, Gibbs distributions, and the Bayesian restoration of images. IEEE Trans Pattern Anal Mach Intell. 1984;6: 721–741. doi: 10.1109/tpami.1984.4767596 22499653

[54] Gilks W, Richardson S, Spiegelhalter D. Markov Chain Monte Carlo in practice. London: Chapman and Hall; 1996.

[55] Purwins S, Herberger K, Debus ES, Rustenbach SJ, Pelzer P, Rabe E, et al. Cost-of-illness of chronic leg ulcers in Germany. Int Wound J. 2010;7: 97–102. doi: 10.1111/j.1742-481X.2010.00660.x 20529149 PMC7951196

[56] Barshes NR, Chambers JD, Cohen J, Belkin M. Cost-effectiveness in the contemporary management of critical limb ischemia with tissue loss. J Vasc Surg. 2012;56: 1015–1024.e1. doi: 10.1016/j.jvs.2012.02.069 22854267

[57] Garrison LP, Neumann PJ, Erickson P, Marshall D, Mullins CD. Using real-world data for coverage and payment decisions: The ISPOR Real-World Data Task Force Report. Value Health. 2007;10: 326–335. doi: 10.1111/j.1524-4733.2007.00186.x 17888097

[58] Gilligan AM. Health economics and outcomes research of wound care: overview of methodology. Adv Wound Care. 2018;7: 380–386. doi: 10.1089/wound.2018.0817 30479871 PMC6247976

[59] Briggs AH, Claxton K, Sculpher MJ. Decision modelling for health economic evaluation. Oxford: Oxford University Press; 2006.

[60] Briggs AH, Gray AM. Handling uncertainty when performing economic evaluation of healthcare interventions. Health Technol Assess (Rockv). 1999;3. 10448202

