# Supplementary material for: Cost-effectiveness analysis of domiciliary topical sevoflurane for painful leg ulcers
Source: PLoS One. 2021 Sep 20;16(9):e0257494. doi: 10.1371/journal.pone.0257494 (PMC8452083; doi:10.1371/journal.pone.0257494)
Supplement: S1 File — (PDF) [file pone.0257494.s001.pdf]

S1 File. Background on the use of topical sevoflurane

Patients suffering from refractory painful caused by nonrevascularizable vascular chronic leg ulcers were referred to a Pain Clinic for specialized pain management. Usual analgesic treatment was mainly based on conventional systemic analgesic drugs (non-steroidal anti-inflammatory drugs, opioids, and adjuvants). In addition to conventional analgesics, patients not presenting any safety concern -mainly having small children living in the same house, or suffering from mental disorders-, were offered to be treated with *off-label* domiciliary topical sevoflurane following a specific protocol.

This protocol was approved by our Institutional Review Board and by the Off-Label and Rare Diseases Subcommittee depending of the Pharmacy Committee (Code CFF-SEV-2013-01, “Prospective observational study to assess a protocol of off-label topical sevoflurane on vascular chronic ulcers”). It was also classified by the Spanish Agency of Drugs and Sanitary Products as a ”Post-market study with a design different to prospective follow-up”. Patients had to sign an informed consent to be treated.

Briefly, the protocol is as follows. Upon acceptance, leg ulcers are irrigated with topical sevoflurane at a dose of approximately 1mL / cm^2 (areas are calculated by the Kundin method, Area = Length × Width × 0.785).

The first irrigation is always performed at the Pain Clinic using prefilled syringes charged with liquid sevoflurane, which are provided by the Department of Pharmacy. Then, patients and their relatives were instructed on how to apply sevoflurane at home and they were given enough syringes for the period between scheduled visits to the Pain Clinic in a monthly/bimonthly basis, depending on the case.

All patients attended at the pain clinic, regardless of the cause, are asked at every visit to rate their pain intensity using the Numerical Rate Scale (NRS), which ranges from 0 (no pain at all) to 10 (the worst imaginable pain).
