# Supplementary material for: Cost-effectiveness analysis of domiciliary topical sevoflurane for painful leg ulcers
Source: PLoS One. 2021 Sep 20;16(9):e0257494. doi: 10.1371/journal.pone.0257494 (PMC8452083; doi:10.1371/journal.pone.0257494)
Supplement: S2 File — (PDF) [file pone.0257494.s002.pdf]

S2 File. Selection of covariates for Bayesian regression model

Multiple linear regression analysis was performed to examine the relationship between potential predictive factors and dependent variables (costs and effectiveness). Covariates to be included in a linear regression model were selected after a three-step process. First, level of association between all baseline variables (age; sex; weight; diabetes mellitus; arterial hypertension; ulcer etiology; ulcer duration; ulcers per patient; ulcer depth; ulcer area; ulcer-related pain; neuropathic pain; analgesics, number of active principles; patients taking major opioids only (%); patients taking major or minor opioids (%); OME only for patients taking opioids (mg/d) was explored using Spearman correlation test.

Variables with high correlations (r>|±0·4|) were previously excluded from analyses. Second, the eventual relationship between each dependent variable and all independent basal variables was explored through a simple linear regression, and those covariates reaching a level of significance less than or equal to 0.1 for any of the two dependent variables were chosen for the exploratory multivariate analysis (see table below). Third, these covariates were included in the multivariate regression model, using the backward elimination method. Finally, covariates for the definitive multiple regression analysis were selected according to both statistical and clinical criteria.

Table showing the linear regression coefficient analysis with Costs and Effectiveness (SPID) as dependent outcome variables.

	**Costs**				**Effectiveness (SPID)**			
	**ß**	**LL CI95%**	**UL CI95%**	**p-value**	**ß**	**LL CI95%**	**UL CI95%**	**p-value**
Age	-106.76	-404.77	191.26	0.477	-0.15	-0.60	0.30	0.508

Sex/Gender	-6402.19	-13311.32	506.94	0.069	-2.44	-13.09	8.21	0.648
Weight	95.40	-131.86	322.66	0.405	-0.20	-0.54	0.14	0.244
Diabetes mellitus	4824.25	-2706.65	12355.14	0.205	-5.48	-16.86	5.90	0.339
Arterial hypertension	8716.09	1139.94	16292.25	**0.025**	0.08	-11.78	11.94	0.989
Ischemic etiology of the ulcer	7141.30	-7145.11	21427.71	0.322	2.48	-19.15	24.11	0.820
Duration of the ulcer	170.82	46.40	295.24	**0.008**	0.19	-0.01	0.38	0.053
Number of ulcers per patient	3513.88	967.89	6059.87	**0.008**	-0.41	-4.46	3.65	0.842
Depth beyond the dermis (Y/N)	9907.75	2573.86	17241.64	**0.009**	4.187	-7.41	15.79	0.473
Area of the main ulcer	403.88	88.78	718.99	**0.013**	0.11	-0.39	0.61	0.654
Baseline pain (NRS)	872.33	-1466.27	3210.93	0.459	4.17	0.80	7.54	**0.016**
Neuropathic pain (Y/N)	4428.58	-2507.86	11365.02	0.207	-2.86	-13.39	7.67	0.589
Number of daily analgesics, active principles	415.97	-2760.66	3592.60	0.794	-0.46	-5.24	4.31	0.848
Patients taking opioids (Y/N)	1372.34	-8649.16	11393.85	0.785	-4.37	-19.40	10.65	0.563
Baseline OME	11.07	-30.11	52.24	0.593	-0.01	-0.07	0.06	0.890
Treatment with sevoflurane (Y/N)	-3544.09	-10584.55	3496.38	0.318	28.36	20.50	36.23	**<0.001**

LL: Lower limit. UL: Upper limit. CI95%: 95% confidence interval. SPID: Summed pain Intensity Differences (see main text for details). NRS: Numerical Rating Scale. Y/N: Yes/No. OME: Oral morphine equivalent, in mg.
