# Supplementary material for: Cost-effectiveness analysis of domiciliary topical sevoflurane for painful leg ulcers
Source: PLoS One. 2021 Sep 20;16(9):e0257494. doi: 10.1371/journal.pone.0257494 (PMC8452083; doi:10.1371/journal.pone.0257494)
Supplement: S3 File — (PDF) [file pone.0257494.s003.pdf]

S3 File. OpenBUGS codes for cost-effectiveness analysis

```
#CEA-Model{

for(i in 1:N){
output[i,1] <- effectiveness[i]
output[i,2] <- log(cost[i])
output[i,1:2] ~ dmnorm(mu[i,1:2],v[1:2,1:2])
mu[i,1] <- beta1[1] + beta1[2]*AHT[i] + beta1[3]*UlcerDuration[i]+beta1[4]*UlcerNumber[i] + beta1[5]*UlcerDepth[i] + beta1[6]*UlcerPain[i]+beta1[7]*Treatment[i]
mu[i,2] <- beta2[1] + beta2[2]*AHT[i] + beta2[3]*UlcerDuration[i]+beta2[4]*UlcerNumber[i] + beta2[5]*UlcerDepth[i] + beta2[6]*UlcerPain[i]+beta2[7]*Treatment[i]}

beta1[1:7] ~ dmnorm(a[], A[,])
beta2[1:7] ~ dmnorm(b[], B[,])
v[1:2,1:2] ~ dwish(A1[,],f1)

#Mean effectiveness.
EffectivenessMeanT1 <- beta1[1] + beta1[2]*0.7344 + beta1[3]*21.3438+beta1[4]*2.0469 + beta1[5]*0.2813 + beta1[6]*6.7813+ beta1[7]
EffectivenessMeanT0 <-beta1[1] + beta1[2]*0.7344 + beta1[3]*21.3438 + beta1[4]*2.0469 + beta1[5]*0.2813 + beta1[6]*6.7813

#Mean cost.
Var[1:2,1:2]<-inverse(v[1:2,1:2])

mumedioT1<-beta2[1] + beta2[2]*0.7344 + beta2[3]*21.3438+beta2[4]*2.0469 + beta2[5]*0.2813 + beta2[6]*6.7813 + beta2[7]
MeanCostT1 <- exp(mumedioT1+Var[2,2]/2)
mumedioT0<-beta2[1] + beta2[2]*0.7344 + beta2[3]*21.3438+beta2[4]*2.0469 + beta2[5]*0.2813 + beta2[6]*6.7813
MeanCostT0 <- exp(mumedioT0+Var[2,2]/2)

# Incremental effectiveness and incremental cost.
DeltaE <- beta1[7]
ratioC <- exp(beta2[7])

# Cost-effectiveness acceptability curve.
for (k in 1:NK){
```

```
Q[k] <- step(Rc[k]*DeltaE - (ratioC-1))}

# Probabilities.
effec <- step(beta1[7])
cheap <- 1-step(exp(beta2[7])-1)
dominant <- effec*cheap}

# Priors (non-informative).
list(a=c(0,0,0,0,0,0,0), A=structure(.Data =
c(0.00001,0,0,0,0,0,0,0,0.00001,0,0,0,0,0,0,0,0.00001,0,0,0,0,0,0,0,0.00001,0,0,0,0,0,0,0,0.00001,0,0,0,0,
0,0,0,0.00001,0,0,0,0,0,0,0,0.00001),.Dim=c(7,7)),
b=c(0,0,0,0,0,0,0), B=structure(.Data =
c(0.00001,0,0,0,0,0,0,0,0.00001,0,0,0,0,0,0,0,0.00001,0,0,0,0,0,0,0,0.00001,0,0,0,0,0,0,0,0.00001,0,0,0,0,
0,0,0,0.00001,0,0,0,0,0,0,0,0.00001), .Dim=c(7,7)), A1=structure(.Data =
c(1,0,0,1),.Dim=c(2,2)), f1=2)

#Data.
list(N=64)
Treatment[]cost[]effectiveness[]AHT[]UlcerDuration[]UlcerNumber[]UlcerDepth[]UlcerPain[]
1	9161	38	0	72	4	0	8
1	697	16.7	0	3	1	0	7
(…60 more rows)
0	19305	4.7	1	12	1	0	6
0	1084	9.1	1	14	3	0	6
END

# Rc values.
list(NK=10, Rc=c(0,10,20,30,40,50,60,70,80,90))

# Starting values.
list(v=structure(.Data=c(1,0,0,1),.Dim=c(2,2)), beta1=c(1,1,1,1,1,1,1), beta2=c(1,1,1,1,1,1,1))
```
