# Supplementary material for: Cost-effectiveness analysis of domiciliary topical sevoflurane for painful leg ulcers
Source: PLoS One. 2021 Sep 20;16(9):e0257494. doi: 10.1371/journal.pone.0257494 (PMC8452083; doi:10.1371/journal.pone.0257494)
Supplement: S2 Table — (PDF) [file pone.0257494.s005.pdf]

**S2 Table. Mean consumption of pharmaceutical formulations for each group.**

| | SEVOFLURANE (n = 38) | CONVENTIONAL (n = 26) | UNIT COSTS (€) |
|---|---|---|---|
| **OPIOIDS** | | | |
| Buprenorphine TD 35 μg | 1.8 | 0.7 | 21.54 |
| Buprenorphine TD 52.5 μg | 0.0 | 5.3 | 32.3 |
| Buprenorphine TD 70 μg | 9.1 | 13.1 | 43.07 |
| Codeine 30 mg | 10.9 | 22.7 | 2.34 |
| Fentanyl TD 25 μg | 6.4 | 3.1 | 14.63 |
| Fentanyl TD 50 μg | 3.0 | 7.7 | 29.25 |
| Fentanyl TD 75 μg | 2.9 | 2.1 | 43.88 |
| Fentanyl TD 100 μg | 1.2 | 5.9 | 58.51 |
| Hydromorphone 8 mg | 6.5 | 0.0 | 32.38 |
| Hydromorphone 16 mg | 8.1 | 0.0 | 64.75 |
| Hydromorphone 32 mg | 0.9 | 0.0 | 129.51 |
| Morphine 20 mg | 6.8 | 21.2 | 3.67 |
| Morphine 30 mg | 0.0 | 39.2 | 26.91 |
| Morphine 60 mg | 17.3 | 50.4 | 49.36 |
| Morphine 90 mg | 1.8 | 0.0 | 35.81 |
| Oxycodone 10 mg | 12.6 | 0.0 | 25.38 |
| Oxycodone 20 mg | 8.6 | 22.9 | 50.77 |
| Oxycodone 40 mg | 28.6 | 13.8 | 101.53 |
| Oxycodone 80 mg | 0.0 | 15.3 | 60.88 |
| Tapentadol 100 mg | 24.1 | 0.0 | 88.05 |
| Tapentadol 150 mg | 3.3 | 25.2 | 132.07 |
| Tapentadol 200 mg | 12.2 | 58.0 | 153.33 |
| Tramadol 37.5 mg (paracetamol 325 mg) | 58.9 | 41.2 | 8.88 |
| Tramadol 75 mg (paracetamol 650 mg) | 29.0 | 21.5 | 17.77 |
| Tramadol 50 mg | 33.4 | 0.0 | 6.21 |

| | | | |
|---|---|---|---|
| Tramadol 100 mg | 8.8 | 0.0 | 12.41 |
| Tramadol 150 mg | 23.9 | 28.4 | 18.62 |
| Tramadol 200 mg | 3.4 | 14.0 | 24.82 |
| **NONOPIOIDS** | | | |
| Amitriptyline 10 mg | 3.6 | 0.0 | 1.06 |
| Amitriptyline 25 mg | 33.1 | 12.9 | 1.56 |
| Clonazepam 2 mg | 3.6 | 0.0 | 2.7 |
| Dexketoprofen 25 mg | 16.3 | 4.9 | 4 |
| Diazepam 5 mg | 7.4 | 0.0 | 1.5 |
| Diclofenac 75 mg | 3.4 | 8.5 | 2.5 |
| Etoricoxib 60 mg | 0.0 | 4.3 | 10.74 |
| Etoricoxib 90 mg | 6.6 | 23.6 | 16.11 |
| Gabapentin 300 mg | 31.4 | 95.3 | 9.23 |
| Gabapentin 400 mg | 29.4 | 0.0 | 12.3 |
| Gabapentin 600 mg | 43.9 | 69.0 | 18.45 |
| Ibuprofen 400 mg | 4.0 | 21.6 | 1.97 |
| Ibuprofen 600 mg | 19.3 | 53.3 | 2.06 |
| Metamizole 575 mg | 237.9 | 466.3 | 2.26 |
| Metamizole 2 g | 22.1 | 0.0 | 2.14 |
| Nabumetone 1 g | 26.0 | 60.5 | 10.82 |
| Paracetamol 650 mg | 34.1 | 82.6 | 1.31 |
| Paracetamol 1g | 225.8 | 223.0 | 1.9 |
| Pregabalin 25 mg | 0.0 | 25.5 | 4.9 |
| Pregabalin 50 mg | 31.9 | 61.5 | 9.8 |
| Pregabalin 75 mg | 90.3 | 107.0 | 14.71 |
| Pregabalin 150 mg | 36.8 | 99.1 | 29.41 |
| Pregabalin 300 mg | 0.0 | 9.2 | 58.82 |

TD, transdermal.
