# Supplementary material for: Cost-effectiveness analysis of domiciliary topical sevoflurane for painful leg ulcers
Source: PLoS One. 2021 Sep 20;16(9):e0257494. doi: 10.1371/journal.pone.0257494 (PMC8452083; doi:10.1371/journal.pone.0257494)
Supplement: S3 Table — (PDF) [file pone.0257494.s006.pdf]

S3 Table. Sensitivity analyses: Statistical summary of costs and effectiveness (100,000 simulations MCMC)

	SEVOFLURANE		CONVENTIONAL		Incremental difference	
	Mean (SD)	95% CrI	Mean (SD)	95% CrI	Mean (SD)	95% CrI
**No patient suffered from complications while admitted**						
**Costs (€)**	10560 (2275)	(7515; 14700)	18330 (4597)	(12280; 26770)	0.60 (0.17)	**(0.37; 0.92)**
**Effectiveness (SPID)**	41.37 (2.32)	(37.56; 45.17)	13.23 (2.82)	(8.59; 17.86)	28.15 (3.70)	**(22.07; 34.22)**
**All patients suffered from complications while admitted**						
**Costs (€)**	10670 (2350)	(7546; 14940)	21570 (5517)	(14340; 31680)	0.52 (0.15)	**(0.31; 0.79)**
**Effectiveness (SPID)**	41.37 (2.32)	(37.56; 45.16)	13.22 (2.82)	(8.58; 17.86)	28.15 (3.70)	**(22.06; 34.23)**
**Admissions attributed to the ulcers**						
**Costs (€)**	6849 (1323)	(5043; 9248)	9599 (2164)	(6678; 13540)	0.74 (0.19)	**(0.47; 1.10)**
**Effectiveness (SPID)**	41.37 (2.32)	(37.55; 45.17)	13.22 (2.82)	(8.58; 17.85)	28.15 (3.70)	**(22.07; 34.25)**
**Admissions attributed to the pain caused by the ulcers**						
**Costs (€)**	5717 (989)	(4338; 7499)	7064 (1435)	(5089; 9672)	0.84 (0.20)	**(0.55; 1.20)**
**Effectiveness (SPID)**	41.37 (2.32)	(37.56; 45.16)	13.22 (2.82)	(8.58; 17.86)	28.15 (3.70)	**(22.06; 34.23)**
**Costs of admittance calculated by multiplying the cost attributed to every day of hospitalization by the length of stay**						
**Costs (€)**	7865 (1332)	(5827; 10080)	11650 (2371)	(8385; 15960)	0.68 (0.16)	**(0.45; 0.98)**
**Effectiveness (SPID)**	41.37 (2.32)	(37.56; 45.16)	13.22 (2.82)	(8.58; 17.86)	28.15 (3.70)	**(22.06; 34.22)**
**Excluding 11 patients who experienced a negative outcome (8 patients died, 3 legs were amputated)**						
**Costs (€)**	8205 (1915)	(5713; 11680)	15870 (4402)	(10240; 23910)	0.55 (0.17)	**(0.32; 0.86)**

**Effectiveness (SPID)**	40.62 (2.45)	(36.60; 44.63)	14.08 (3.03)	(9.10; 19.07)	26.54 (3.98)	**(20.01; 33.08)**
**Extreme scenario analysis (worst scenario for SEVOFLURANE)**						
**Costs (€)**	12820 (2742)	(9146; 17800)	14810 (3685)	(9945; 21560)	0.91 (0.26)	**(0.55; 1.38)**
**Effectiveness (SPID)**	30.94 (2.01)	(27.64; 34.22)	16.60 (2.44)	(12.58; 20.60)	14.34 (3.19)	**(9.09; 19.60)**

Intervals not including the zero value are highlighted in bold. CrI, Credible Interval; MCMC, Markov Chain Monte Carlo; SPID, Summed Pain Intensity Differences.
