# Supplementary material for: Cost-effectiveness analysis of domiciliary topical sevoflurane for painful leg ulcers
Source: PLoS One. 2021 Sep 20;16(9):e0257494. doi: 10.1371/journal.pone.0257494 (PMC8452083; doi:10.1371/journal.pone.0257494)
Supplement: S4 Table — (PDF) [file pone.0257494.s007.pdf]

S4 Table. Sensitivity analyses: Estimations of the posterior distribution of the β-coefficients and of the probabilities related to the cost-effectiveness analysis (100,000 simulations MCMC)

**No patient suffered from complications while admitted**		**Mean (SD)**	**95% CrI**
**Costs**	β_{11} *intercept*	7.45 (0.74)	**(6.24; 8.66)**
	β_{12} *AHT*	0.43 (0.31)	(-0.09; 0.94)
	β_{13} *UlcerDuration*	0.02 (0.01)	**(0.01; 0.03)**
	β_{14} *UlcerNumber*	0.29 (0.11)	**(0.11; 0.46)**
	β_{15} *UlcerDepth*	0.94 (0.32)	**(0.40; 1.47)**
	β_{16} *UlcerPain*	0.04 (0.09)	(-0.12; 0.19)
	β_{17} *Treatment*	-0.54 (0.28)	**(-1.00; -0.09)**
	Costs ratio (exp β_{17})	0.60 (0.17)	**(0.37; 0.92)**
**Effectiveness**	β_{21} *intercept*	-22.44 (9.79)	**(-38.43; -6.30)**
	β_{22} *AHT*	1.38 (4.19)	(-5.51; 8.25)
	β_{23} *UlcerDuration*	0.12 (0.07)	**(0.00; 0.23)**
	β_{24} *UlcerNumber*	0.60 (1.42)	**(-1.74; 2.94)**
	β_{25} *UlcerDepth*	1.33 (4.33)	(-5.79; 8.43)
	β_{26} *UlcerPain*	4.51 (1.26)	**(2.44; 6.58)**
	β_{27} *Treatment*	28.15 (3.70)	**(22.07; 34.22)**
**Estimated probability for SEVOFLURANE being less expensive**		0.97 (0.16)	(1.0; 1.0)
**Estimated probability for SEVOFLURANE being more effective**		1.0 (0.0)	(1.0; 1.0)
**Estimated probability for SEVOFLURANE being dominant**		0.97 (0.16)	(1.0; 1.0)
**All patients suffered from complications while admitted**		**Mean (SD)**	**95% CrI**

**Costs**	β_{11} *intercept*	7.73 (0.75)	**(6.50; 8.96)**
	β_{12} *AHT*	0.44 (0.32)	(-0.09; 0.96)
	β_{13} *UlcerDuration*	0.02 (0.01)	**(0.01; 0.03)**
	β_{14} *UlcerNumber*	0.29 (0.11)	**(0.12; 0.47)**
	β_{15} *UlcerDepth*	0.97 (0.33)	**(0.42; 1.51)**
	β_{16} *UlcerPain*	0.01 (0.10)	(-0.15; 0.17)
	β_{17} *Treatment*	-0.70 (0.28)	**(-1.16; -0.23)**
	Costs ratio (exp β_{17})	0.52 (0.15)	**(0.31; 0.79)**
**Effectiveness**	β_{21} *intercept*	-22.44 (9.79)	**(-38.40; -6.32)**
	β_{22} *AHT*	1.38 (4.19)	(-5.51; 8.25)
	β_{23} *UlcerDuration*	0.12 (0.07)	**(0.00; 0.23)**
	β_{24} *UlcerNumber*	0.60 (1.42)	(-1.75; 2.95)
	β_{25} *UlcerDepth*	1.33 (4.33)	(-5.78; 8.43)
	β_{26} *UlcerPain*	4.51 (1.26)	**(2.44; 6.58)**
	β_{27} *Treatment*	28.15 (3.70)	**(22.06; 34.23)**
**Estimated probability for SEVOFLURANE being less expensive**		0.99 (0.08)	(1.0; 1.0)
**Estimated probability for SEVOFLURANE being more effective**		1.0 (0.0)	(1.0; 1.0)
**Estimated probability for SEVOFLURANE being dominant**		0.99 (0.08)	(1.0; 1.0)
**Admissions attributed to the ulcers**		**Mean (SD)**	**95% CrI**
**Costs**	β_{11} *intercept*	7.03 (0.68)	**(5.92; 8.15)**
	β_{12} *AHT*	0.20 (0.29)	(-0.28; 0.67)
	β_{13} *UlcerDuration*	0.01 (0.00)	**(0.01; 0.02)**
	β_{14} *UlcerNumber*	0.29 (0.10)	**(0.13; 0.45)**
	β_{15} *UlcerDepth*	0.89 (0.30)	**(0.39; 1.38)**
	β_{16} *UlcerPain*	0.05 (0.09)	(-0.09; 0.19)
	β_{17} *Treatment*	-0.33 (0.26)	(-0.75; 0.09)

	Costs ratio (exp β_{17})	0.74 (0.19)	**(0.47; 1.10)**
**Effectiveness**	β_{21} *intercept*	-22.44 (9.79)	**(-38.42; -6.33)**
	β_{22} *AHT*	1.38 (4.19)	(-5.52; 8.26)
	β_{23} *UlcerDuration*	0.12 (0.07)	**(0.00; 0.23)**
	β_{24} *UlcerNumber*	0.60 (1.43)	(-1.75; 2.94)
	β_{25} *UlcerDepth*	1.33 (4.33)	(-5.78; 8.43)
	β_{26} *UlcerPain*	4.51 (1.26)	**(2.44; 6.57)**
	β_{27} *Treatment*	28.15 (3.70)	**(22.07; 34.25)**
**Estimated probability for SEVOFLURANE being less expensive**		0.90 (0.30)	(0.0; 1.0)
**Estimated probability for SEVOFLURANE being more effective**		1.0 (0.0)	(1.0; 1.0)
**Estimated probability for SEVOFLURANE being dominant**		0.90 (0.30)	(0.0; 1.0)
**Admissions attributed to the pain caused by the ulcers**		**Mean (SD)**	**95% CrI**
**Costs**	β_{11} *intercept*	6.70 (0.63)	**(5.67; 7.73)**
	β_{12} *AHT*	0.29 (0.27)	(-0.15; 0.72)
	β_{13} *UlcerDuration*	0.01 (0.00)	**(0.01; 0.02)**
	β_{14} *UlcerNumber*	0.24 (0.09)	**(0.09; 0.39)**
	β_{15} *UlcerDepth*	0.84 (0.28)	**(0.38; 1.29)**
	β_{16} *UlcerPain*	0.08 (0.08)	(-0.06; 0.21)
	β_{17} *Treatment*	-0.21 (0.24)	(-0.59; 0.18)
	Costs ratio (exp β_{17})	0.84 (0.20)	**(0.55; 1.20)**
**Effectiveness**	β_{21} *intercept*	-22.44 (9.79)	**(-38.40; -6.32)**
	β_{22} *AHT*	1.38 (4.19)	(-5.52; 8.25)
	β_{23} *UlcerDuration*	0.12 (0.07)	**(0.00; 0.23)**
	β_{24} *UlcerNumber*	0.60 (1.42)	(-1.75; 2.95)
	β_{25} *UlcerDepth*	1.33 (4.33)	(-5.78; 8.43)

	β_{26} *UlcerPain*	4.51 (1.26)	**(2.44; 6.58)**
	β_{27} *Treatment*	28.15 (3.70)	**(22.06; 34.23)**
**Estimated probability for SEVOFLURANE being less expensive**		0.81 (0.39)	(0.0; 1.0)
**Estimated probability for SEVOFLURANE being more effective**		1.0 (0.0)	(1.0; 1.0)
**Estimated probability for SEVOFLURANE being dominant**		0.81 (0.39)	(0.0; 1.0)
**Costs of admittance calculated by multiplying the cost attributed to every day of hospitalization by the length of stay**		**Mean (SD)**	**95% CrI**
	β_{11} *intercept*	7.26 (0.63)	**(6.23; 8.29)**
	β_{12} *AHT*	0.45 (0.27)	**(0.01; 0.89)**
	β_{13} *UlcerDuration*	0.02 (0.00)	**(0.01; 0.03)**
**Costs**	β_{14} *UlcerNumber*	0.31 (0.09)	**(0.16; 0.46)**
	β_{15} *UlcerDepth*	0.80 (0.28)	**(0.35; 1.26)**
	β_{16} *UlcerPain*	0.01 (0.08)	(-0.12; 0.15)
	β_{17} *Treatment*	-0.41 (0.24)	**(-0.80; -0.02)**
	Costs ratio (exp β_{17})	0.68 (0.16)	**(0.45; 0.98)**
	β_{21} *intercept*	-22.44 (9.79)	**(-38.39; -6.32)**
	β_{22} *AHT*	1.38 (4.19)	(-5.51; 8.25)
	β_{23} *UlcerDuration*	0.12 (0.07)	**(0.00; 0.23)**
**Effectiveness**	β_{24} *UlcerNumber*	0.60 (1.42)	(-1.75; 2.95)
	β_{25} *UlcerDepth*	1.33 (4.33)	(-5.78; 8.44)
	β_{26} *UlcerPain*	4.51 (1.26)	**(2.44; 6.58)**
	β_{27} *Treatment*	28.15 (3.70)	**(22.06; 34.22)**
**Estimated probability for SEVOFLURANE being less expensive**		0.96 (0.20)	(1.0; 1.0)

**Estimated probability for SEVOFLURANE being more effective**		1.0 (0.0)	(1.0; 1.0)
**Estimated probability for SEVOFLURANE being dominant**		0.96 (0.20)	(1.0; 1.0)
**Excluding 11 patients who experienced a negative outcome (8 patients died, 3 legs were amputated)**		**Mean (SD)**	**95% CrI**
**Costs**	β_{11} *intercept*	7.51 (0.78)	**(6.22; 8.80)**
	β_{12} *AHT*	0.53 (0.33)	(-0.01; 1.08)
	β_{13} *UlcerDuration*	0.02 (0.01)	**(0.01; 0.03)**
	β_{14} *UlcerNumber*	0.28 (0.11)	**(0.10; 0.47)**
	β_{15} *UlcerDepth*	0.72 (0.39)	**(0.09; 1.35)**
	β_{16} *UlcerPain*	0.02 (0.10)	(-0.15; 0.18)
	β_{17} *Treatment*	-0.65 (0.30)	**(-1.15; -0.15)**
	Costs ratio (exp β_{17})	0.55 (0.17)	**(0.32; 0.86)**
**Effectiveness**	β_{21} *intercept*	-25.77 (10.25)	**(-42.53; -8.86)**
	β_{22} *AHT*	1.05 (4.37)	(-6.12; 8.26)
	β_{23} *UlcerDuration*	0.11 (0.07)	(-0.00; 0.22)
	β_{24} *UlcerNumber*	0.56 (1.48)	(-1.86; 2.98)
	β_{25} *UlcerDepth*	6.78 (5.07)	(-1.51; 15.08)
	β_{26} *UlcerPain*	5.18 (1.28)	**(3.08; 7.29)**
	β_{27} *Treatment*	26.54 (3.98)	**(20.01; 33.08)**
**Estimated probability for SEVOFLURANE being less expensive**		0.98 (0.13)	(1.0; 1.0)
**Estimated probability for SEVOFLURANE being more effective**		1.0 (0.0)	(1.0; 1.0)
**Estimated probability for SEVOFLURANE being dominant**		0.98 (0.13)	(1.0; 1.0)

Extreme scenario analysis (worst scenario for SEVOFLURANE)		Mean (SD)	95% CrI
**Costs**	β_{11} *intercept*	7.36 (0.73)	**(6.16; 8.57)**
	β_{12} *AHT*	0.43 (0.31)	(-0.09; 0.93)
	β_{13} *UlcerDuration*	0.02 (0.01)	**(0.01; 0.03)**
	β_{14} *UlcerNumber*	0.30 (0.11)	**(0.12; 0.47)**
	β_{15} *UlcerDepth*	0.96 (0.32)	**(0.42; 1.49)**
	β_{16} *UlcerPain*	0.01 (0.09)	(-0.14; 0.17)
	β_{17} *Treatment*	-0.14 (0.28)	(-0.59, 0.32)
	Costs ratio (exp β_{17})	0.91 (0.26)	**(0.55; 1.38)**
**Effectiveness**	β_{21} *intercept*	-12.83 (8.46)	(-26.65; 1.09)
	β_{22} *AHT*	0.46 (3.62)	(-5.50; 6.40)
	β_{23} *UlcerDuration*	0.09 (0.06)	(-0.01; 0.19)
	β_{24} *UlcerNumber*	-0.30 (1.23)	(-2.32; 1.73)
	β_{25} *UlcerDepth*	0.54 (3.74)	(-5.59; 6.68)
	β_{26} *UlcerPain*	4.08 (1.09)	**(2.29; 5.87)**
	β_{27} *Treatment*	14.34 (3.19)	**(9.09; 19.60)**
**Estimated probability for SEVOFLURANE being less expensive**		0.69 (0.46)	(0.0; 1.0)
**Estimated probability for SEVOFLURANE being more effective**		1.0 (0.01)	(1.0; 1.0)
**Estimated probability for SEVOFLURANE being dominant**		0.69 (0.46)	(0.0; 1.0)

Intervals not including the zero value are highlighted in bold. AHT, Arterial Hypertension; CrI, Credible Interval.
