# Supplementary figures and images for: Cost-effectiveness analysis of domiciliary topical sevoflurane for painful leg ulcers
Source: PLoS One. 2021 Sep 20;16(9):e0257494. doi: 10.1371/journal.pone.0257494 (PMC8452083; doi:10.1371/journal.pone.0257494)

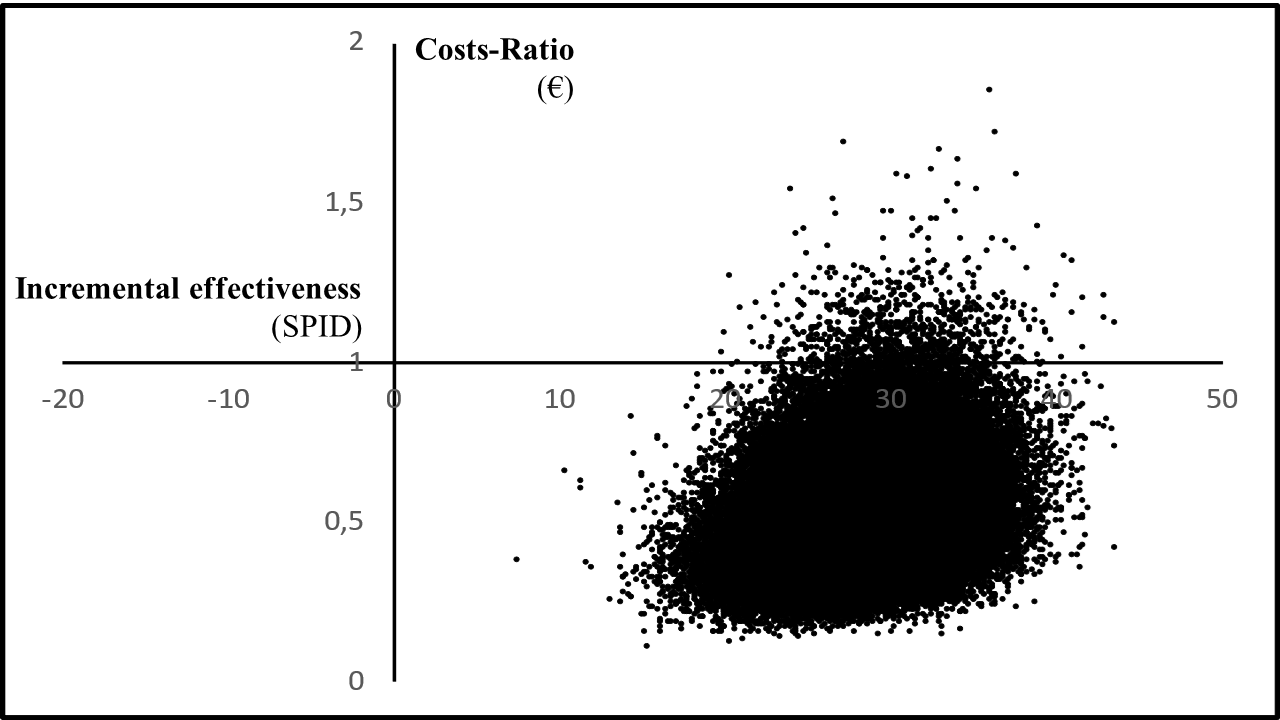

Supplement: S1 Striking image — (TIF) [file pone.0257494.s009.tif]
